# Age‐Like Methylation Changes of HSCs in GADD45B Knockout Mice Define Methylation Sites Associated With Loss of Function

**DOI:** 10.1111/acel.70453

**Published:** 2026-03-20

**Authors:** Wakako Kuribayashi, Mayuri Tanaka‐Yano, Bongsoo Park, Hagai Yanai, Ferda Tekin‐Turhan, Isabel Beerman

**Affiliations:** ^1^ Epigenetics and Stem Cell Aging Unit, TGB National Institute on Aging, NIH Baltimore Maryland USA

**Keywords:** aging, DNA methylation, epigenetics, Gadd45b, hematopoietic stem cell, searchable DNA methylation database

## Abstract

Hematopoietic stem cells (HSCs) self‐renew and differentiate into all blood cells maintaining the hematopoietic system. Age‐related HSC dysfunction impacts all of hematopoiesis, with DNA methylation alterations in aged HSCs contributing to altered function. Growth Arrest and DNA Damage‐inducible proteins (*Gadd45a*, *Gadd45b*, and *Gadd45g*) are expressed in HSC activation, and *Gadd45b* has been reported to induce DNA demethylation. Thus, we explored the relationship between *Gadd45b*, DNA methylation and age‐related HSC changes. WGBS on HSCs from GADD45B knockout mice demonstrated young knockout HSCs have increased DNA methylation, with both unique and overlapping methylation changes compared to aged wild‐type HSCs without reflecting aging transcriptional changes. Peripheral blood and bone marrow analysis, competitive transplants, and single‐cell culture analyses showed no significant loss of functional potential in the aberrantly methylated GADD45B knockout HSCs. We concluded these altered methylation sites don't alter HSC potential. We generated a searchable HSC DNA methylation database incorporating available datasets and present a truncated list of methylation sites associated with changes in HSC function for prioritization to target for resetting the age‐associated loss of HSC potential.

## Graphical abstract

GADD45B knockout in hematopoietic stem cells causes DNA methylation changes that mirror some age‐associated patterns but don't affect HSC function or gene expression. We created a searchable database to identify consistently reported methylation sites associated with age‐related HSC functional decline.

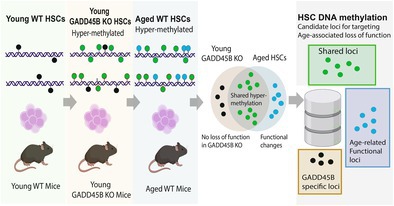

## 1 Introduction

Aging leads to a decline in the functional capacity of hematopoietic stem cells (HSCs), characterized by an increase in HSC numbers and a bias towards myeloid and platelet differentiation in the murine model (Beerman et al. 2010). Changes have been reported in the epigenome and chromatin organization of old HSCs compared to young HSCs (Beerman et al. 2013; Itokawa et al. 2022; Sun et al. 2014). Reported global DNA methylation (DNAm) levels in young HSCs range from 70% to 90% (Cabezas‐Wallscheid et al. 2014; Sun et al. 2014), with DNAm changes potentially influencing their differentiation potentials (Beerman et al. 2013; Yu et al. 2016). The role of methylating and demethylating DNA is performed largely by DNA methyltransferase (DNMT) and ten‐eleven translocation (TET) family proteins (Ambrosi et al. 2017). Murine models of mutant *Dnmt* and *Tet* family genes in hematopoiesis show loss of regulation of the HSC differentiation decisions (Challen et al. 2014; Cimmino et al. 2015; Moran‐Crusio et al. 2011; Tie et al. 2020; Trowbridge et al. 2009) leading to both clonal expansion and altered myeloid cell production. These are also phenotypes associated with aging. In addition, the expression level of *Dnmt3a*, *Dnmt3b*, *Dnmt1*, and *Tet1* decreases in old HSCs compared to young HSCs, suggesting changes in DNAm regulation is also associated with aging (Flohr Svendsen et al. 2021; Sun et al. 2014). Previous studies have reported global DNAm levels in HSCs increase with age (Beerman et al. 2013; Kuribayashi et al. 2021; Sun et al. 2014); however, it is not well understood whether age‐related changes in DNA methylation directly affect functional potential in HSCs.

Old HSCs accumulate DNA damage yet have the capacity to elicit DNA damage response pathways (Beerman et al. 2014; Flach et al. 2014). During quiescence, DNA repair pathways, that include the GADD45 family, are attenuated but activated upon entry into the cell cycle (Beerman et al. 2014). The GADD45 protein family, consisting of GADD45A, GADD45B, and GADD45G, plays a crucial role in stress signaling by activating multiple pathways to enhance cell survival under DNA damage induced stress (Niehrs and Schafer 2012). This critical role in DNA damage is often the focus of this family, but overexpression of *Gadd45a* or *Gadd45b* in nerve cells leads to increased DNA damage as well as induction of DNA demethylation (Barreto et al. 2007; Ravel‐Godreuil et al. 2021). *Gadd45b* has also been implicated in demethylation of brain cells (Labonte et al. 2019; Ma et al. 2009). To reveal if GADD45B plays a role in regulating DNA methylation in the hematopoietic system during aging, we studied a knockout model of GADD45B to assess its role in DNA methylation and functional potential in hematopoietic stem cells (HSC). We found surprising overlap between sites that lose methylation when missing GADD45B or after aging in HSCs, but these overlapping changes in methylation status did not lead to functional changes. Combining our newly generated DNA methylation datasets with previous manuscripts, we created a searchable database defining reproducibly altered DNA methylation sites in aged HSCs. By subtracting the DNA methylation sites that overlap with the GADD45B KO HSCs—with no functional alterations—we define a significantly reduced number of potential DNA sites to target to mitigate age‐associated functional changes in HSCs.

## 2 Methods

### 2.1 Mice

GADD45B knockout mice are JAX #013101, kindly shared by Dr. Mashiko Negishi at NIEHS, and bred and aged at our NIA animal facility. No significant differences were seen between sexes, so both sexes are included in the data sets. CD45.1 mice were purchased from JAX #002014. All experiments were approved under IACUC 469‐TGB‐2025.

### 2.2 Flow Cytometry Analysis

Complete blood counts were collected using Hemavet 950FS. Frequencies of PB cell populations were generated using flow cytometry after ACK treatment and staining with Ter119 (BioLegend, 116232, 1/200), B220 (BioLegend, 103227, 1/200), Mac‐1 (BioLegend, 101224, 1/200), CD3 (BioLegend, 100214, 1/200), and Gr‐1 (BioLegend, 108434, 1/200); with CD45.1 (BioLegend, 110728, 1/100), CD45.2 (BioLegend, 109820, 1/100) included for transplantation experiments. Bone marrow cells from crushed long bones were treated with ACK and stained with lineage [Ter119, B220, Mac‐1, CD3, Gr‐1, IL7ra (BioLegend, 135024, 1/200)], Sca‐1 (BioLegend, 108126, 1/200), c‐Kit (BioLegend, 105808, 1/200), CD34 (eBioscience, 11‐0341‐85, 1/50), Flk2 (BioLegend, 135310, 1/50), CD150 (BioLegend, 115914, 1/200), and FcγRα (eBioscience, 45‐0161‐82, 1/100).

### 2.3 Competitive Transplantation

Pooled BM cells from 2 mice were c‐Kit enriched (Stemcell Technologies) and stained with antibodies listed above. Two hundred HSCs (PI^−^Lin^−^cKit^+^Sca1^+^CD34^−^Flk2^−^CD150^+^) were transplanted into lethally irradiated (9.56 Gy) recipients against 1 × 10^5^ CD45.1 WBM cells. PB analysis was performed at 4‐week intervals.

### 2.4 5‐Fluorouracil (5‐FU) Treatment

5‐FU was administered at 150 mg/kg by intraperitoneal injection, once every 3 weeks, as previously described (Beerman et al. 2013) for 2 cycles. Animals were continuously monitored for general health and body weight, and no discernable side effects were observed.

### 2.5 Flow Cytometry

Data were acquired on BD FACS Fusion, Discover S8, Canto II, and Aria and analyzed using FlowJo Software (Figure S4).

### 2.6 Single HSC Culture Analysis

Individual HSCs were sorted into wells of 96‐well round‐bottom plates. Differentiation media contained Dulbecco's modified Eagle's medium and F‐12 medium supplemented with 10% fetal calf serum, penicillin/streptomycin, 2 mM GlutaMAX, 50 mM 2‐mercaptoethanol, and 10 ng/mL of murine cytokines: stem cell factor, thrombopoietin, Flt3l, IL‐3, erythropoietin, granulocyte macrophage colony‐stimulating factor. Maintenance media was SF‐O3 (Iwai) supplemented with AlbuMax, penicillin/streptomycin, 2 mM GlutaMAX, 50 mM 2‐mercaptoethanol, supplement (Iwai), and mouse stem cell factor, thrombopoietin, and IL‐12 (Beerman et al. 2014). HSCs were cultured at 37°C in a humidified atmosphere with 5% CO_2_. At denoted time points, cell numbers were counted under a microscope. After 14 days of culture, the size of each colony was measured. Click‐iT Plus EdU Alexa Fluor 647 Flow Cytometry Assay Kit was used on 2000 HSCs from three individual mice (*n* = 3) cultured in differentiation media with 10 μM EdU for 18 h.

### 2.7 RNA‐Seq

RNA was purified with TRIzol Reagent and Direct‐zol RNA Microprep. RNA‐seq libraries were constructed using SMART‐Seq v4 Ultra Low Input RNA Kit for Sequencing. A total of 2.1 billion reads were used for transcriptome sequencing, on an average of 80 million reads per sample (YWT *n* = 4, OWT *n* = 12, YKO *n* = 5, OKO *n* = 7) (Table S7). For analysis of transcriptome datasets, we built an index for STAR using the GENCODE M22 reference feature including protein‐coding and non‐coding genes (Dobin et al. 2013). Prior to sequence alignment, we applied trim galore (version 0.4.3) with cutadapt (version 1.12) to remove any unnecessary genomic fragments (e.g., adapter dimers) and low‐quality nucleotide sequences from the raw reads. We mapped adapter trimmed sequencing reads to the mouse reference genome (mm10) using STAR aligner and calculated the raw count using featureCounts software (gene‐level) (Liao et al. 2014). Differentially expressed genes (DEGs) lists were generated with DESeq2 using cutoff: FC > 1.5, FDR < 0.05 (Love et al. 2014; Martin 2011). Differentially expressed genes (DEGs) were defined using cutoff: fc > 1.5 and adjusted *p* value < 0.05 (https://github.com/genomicspark/RNA‐seq_QC_analysis/blob/master/pipe_code/rnaseq_RUV_pairwise_HSC.R). DEGs pathwas were analyzed with IPA (QIAGEN).

### 2.8 WGBS

WGBS sequencing data were generated by Psomagen. Differentially DNAm CpGs (DMCs) were defined with methylkit using cutoff: DNAm difference > 15%, *q* value < 0.05, CpG site ≥ 1, coverage > 10. Differentially methylated regions (DMRs) were defined with metilene using cutoff: DNAm difference ≥ 10%, FDR ≤ 0.05, CpG site ≥ 3, DMCs ≥ 1. DMRs −1000 to +100 bp from the TSS are defined as DMR‐NPs.

### 2.9 Sequence Analysis

Analyses were performed using R (v4.4.1). We used dplyr, openxlsx, dendsort, circlize, ggplot2, VennDiagram and ComplexHeatmap for PCA, Venn, Upset, and heatmap. Aging Signature DNA Methylation Analysis was developed using shiny, DT, ComplexHeatmap, shinythemes and sharing in NIA‐IRP, NIH ShinyApps (https://niairpnih.shinyapps.io/home/) using mm10.

### 2.10 Statistical Analysis

Prism Software (v9; GraphPad) and JMP Software (v17; JMP Statistical Discovery) were used for statistical analyses. Data are presented as mean ± SEM and *p* value < 0.05 was the minimal threshold for significance. The statistical parameters and number of samples are in figure legends. Hierarchal clustering in heatmaps was performed with Euclidean method.

## 3 Results

### 3.1 GADD45B Knockout in Young Mice Increases DNAm of HSCs to Levels Similar to Aged WT HSCs

**FIGURE 1:**
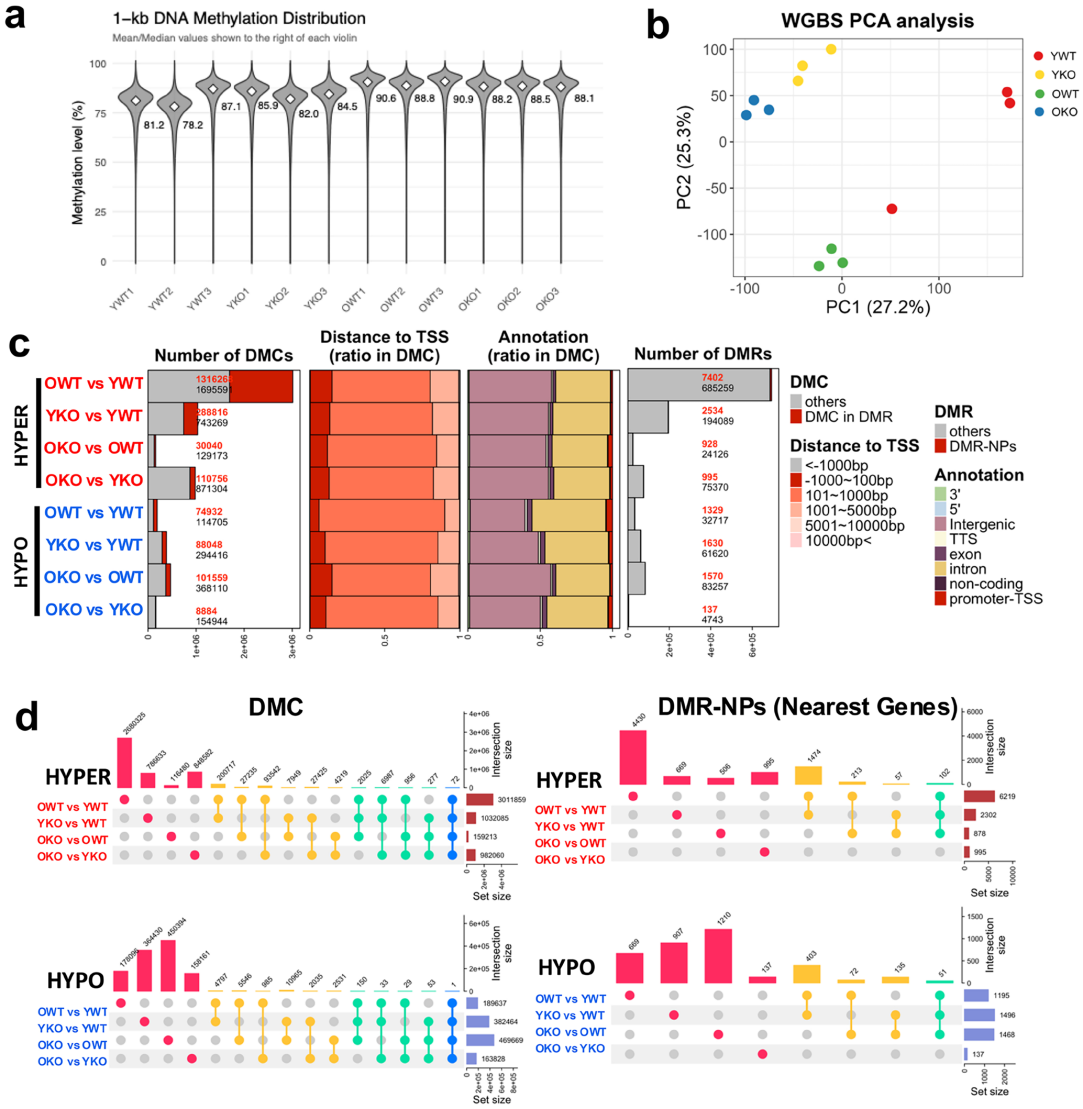
DNA methylation analysis of young and old Gadd45B‐KO and WT HSC (a) Total DNA methylation distribution detected by WGBS analysis of WT and KO HSCs from young and old mice. Value denoted is median global methylation level (b) PCA analysis of WT and KO HSCs from young and old mice using all DNAm values. (c) Number of differentially methylated CpG (DMCs), the proportion of DMCs by relative distance to transcription start site (TSS), the proportion of DMCs per gene location annotation, and number of differentially methylated regions (DMRs) in each comparison of hyper‐ and hypo‐methylation. (d) Upset plot of hyper‐ and hypo‐methylated DMCs and DMRs near promoter regions (DMR‐NPs). The vertical bars indicate the unique events of each combination, and the horizontal bars indicate the total events for each data set comparison.

To test GADD45B's role in DNA demethylation of hematopoietic stem cells (HSC), we performed whole genome bisulfite sequencing on wild‐type (WT) and GADD45B knockout (KO) HSCs isolated from young and old mice. The average global DNA methylation (DNAm) levels of young WT HSCs (YWT) were 82.1%, and these levels were higher (90.1%) in old WT HSCs (OWT) (Figure 1a), concordant with previous reports (Beerman et al. 2013; Kuribayashi et al. 2021; Sun et al. 2014). The same trend of age‐associated methylation increase occurs in the KO HSCs, with higher methylation in the old KO (OKO, 88.2%) compared to young KO (YKO, 84.1%). The YKO also showed higher global levels of methylation compared to YWT, but there was no difference in global methylation levels between OWT and OKO. Principal component analysis (PCA) of DNAm profiles shows OKO and OWT HSCs cluster together, and YKO HSC has methylation patterns more similar to old HSCs' epigenetic landscapes than to YWT (Figure 1b).

To examine the epigenetic modifications driving the PCA clustering, we defined differentially methylated CpGs (DMCs) in comparisons between these groups (Table S1). We established there were over 4 million hyper‐methylated CpGs (3,011,859) and only 189,639 hypo‐methylated DMCs during WT aging (OWT vs. YWT, Figure 1c). Comparisons between YKO and YWT HSCs showed 1,032,085 hyper‐methylated and 382,464 hypo‐methylated DMCs in the young HSCs without GADD45B (Figure 1c). This number of DMCs between the young KO and WT was greater than the number of age‐associated changes between the OKO and YKO (Hyper: 982,060 Hypo: 163,828). Mapping the DMCs to genomic locations showed a quasi‐similar distribution of the locations across all comparisons. Most DMCs were located either > 1 kb upstream or > 10 kb downstream of the TSS (< −1000 bp or 10,000 bp <) and in intronic or intergenic regions. Only a small percentage of DMCs were found near gene promoters. However, when we generated differentially methylated regions (DMRs; Figure S1a, Table S1) the majority of DMRs were located near gene promoter regions (DMR‐NPs, −1000 bp ≤ TSS ≤ 100 bp, Table S2).

To explore specific methylation differences in HSCs without GADD45B and those sites associated with HSC aging, we compared the extent of overlapping methylation changes between the groups. We found the two comparisons that intersected the most frequently were the changes associated with WT aging (OWT vs. YWT) and from loss of GADD45B in young HSCs (YKO vs. YWT), with 209,801 hypermethylated sites (200,717 + 2025 + 6987 + 72) and 5158 hypomethylated sites (4797 + 150 + 33 + 1) overlapping (Figure 1d, Table S3). The second most common overlap was aging‐associated methylation changes, seen in both OWT versus YWT and OKO versus YKO comparisons (Figure 1d). Analysis of methylation levels at DMCs defined from all four comparisons shows hierarchical clustering between the YKO profiles and old HSCs when examining wild‐type age‐associated methylation changes (Figure S1b). Similar clustering (YKO near OWT and OKO) is seen with old HSCs having similar increased methylation compared to YWT at DMCs defined between YKO versus YWT. However, when we evaluate the DMCs of old KO HSCs, the methylation profiles cluster either by GADD45B expression (OKO vs. OWT) or by age (OKO vs. YKO) (Figure S1b). These results suggest DNAm changes induced by the loss of GADD45B generate some methylation changes associated with WT aging, with some of these DNAm changes being maintained during aging in the knockouts; however, there are also aging‐specific and GADD45B‐KO specific DNAm alterations.

We also see a significant enrichment of overlap between altered methylation in the OWT versus YWT compared to YKO versus YWT and with OKO versus YKO when examining DMR‐NPs (Figure 1d) and given this striking overlap between aging and loss of GADD45B in young HSC methylation patterns, we examined whether DNA marks used to measure biological age were also affected. Epigenetic age of the HSCs from the GADD45B‐KO and control mice was calculated using multiple DNAm clocks (Lu et al. 2023; Meer et al. 2018; Petkovich et al. 2017; Stubbs et al. 2017; Thompson et al. 2018), and the biological age of the KO HSCs appeared largely unaffected by these methylation alterations driven by loss of GADD45B (Figure S1c).

### 3.2 Aging‐Associated HSC Transcriptional Alterations Maintained in GADD45B‐KO

**FIGURE 2:**
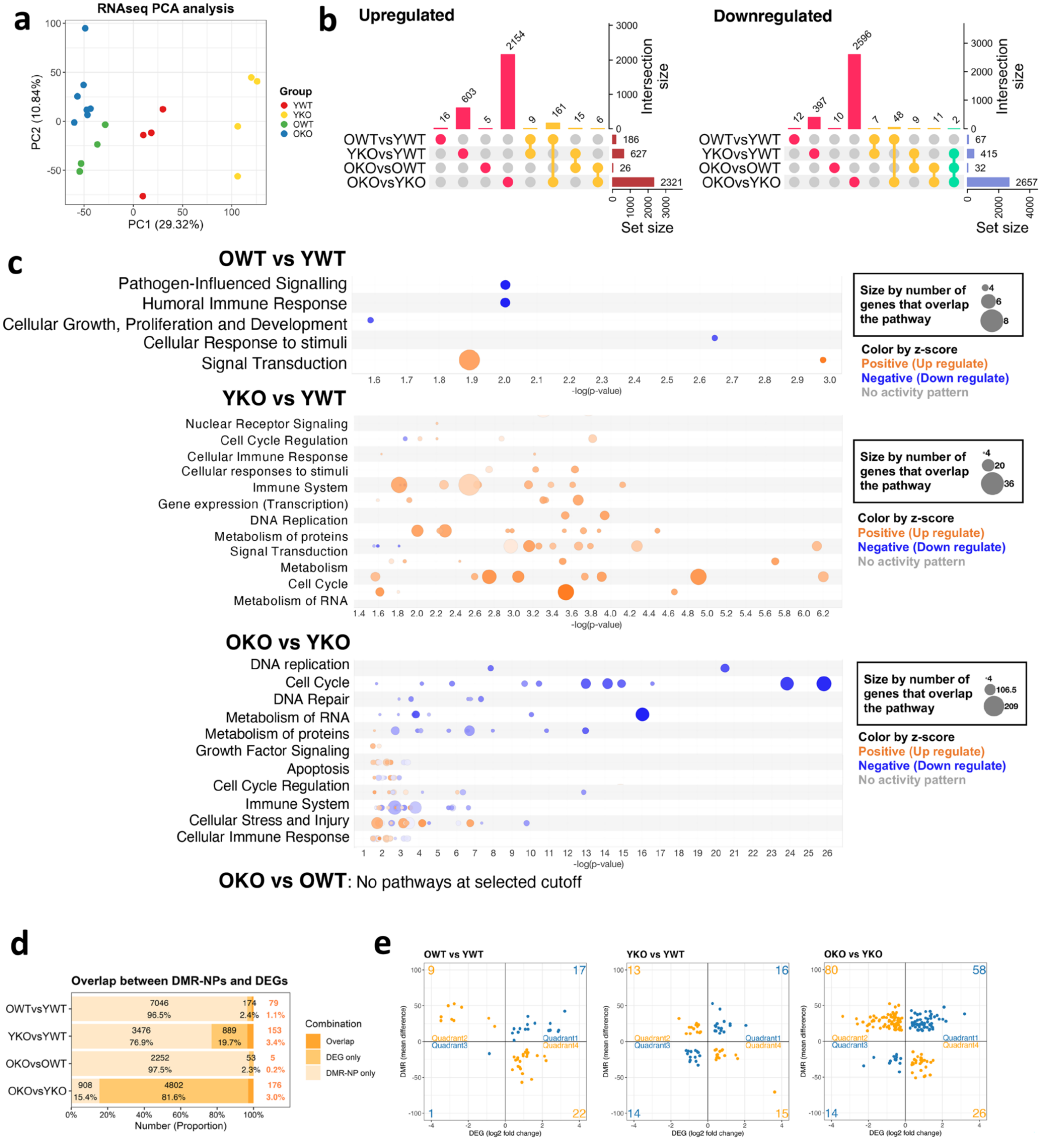
Transcriptional analysis of young and old Gadd45B‐KO and WT HSC (a) PCA analysis of RNA sequencing data of WT and KO HSCs isolated from young and old mice using all data normalized by CPM. (b) Upset plot of up‐ and down‐regulated DEGs. The vertical bars indicate the unique events (differentially expressed gene) at each combination, and the horizontal bars indicate the total number in each comparison. (c) IPA pathway analysis results of DEGs identified in each combination. Orange indicates pathways derived from increased DEGs and blue indicates pathways with decreased DEGs. The color intensity indicates the *z* score, and the size of the circle indicates the number of overlapping genes in the pathway. There were no enriched pathways found in the OKO versus OWT DEGs. (d) Proportion and absolute number of overlaps between methylation (DMR‐NP) and expression (DEG) changes for each comparison. (e) The relationship between DNAm changes (mean of difference) and transcriptome changes (log_2_ fold change) was plotted for genes with overlapping DMR‐NP and DEG. The number of genes belonging to each quadrant is indicated.

To determine whether GADD45B‐KO aging transcriptional signatures were similar or divergent to WT aging‐associated transcriptional changes and if these altered expression profiles corresponded with DNAm changes, we performed RNA sequencing on WT and KO HSCs from young and old mice. PCA shows the gene expression profiles of YKO HSCs separate furthest from the others by PC1, whereas both KO and WT old HSCs clustered closely with each other (Figure 2a). This suggests, in contrast to DNA methylation, transcriptional landscapes of GADD45B‐KO in young HSCs did not overlap with aging phenotypes. To look more explicitly at changes, we defined differentially expressed genes (DEGs) between each comparison with the threshold of adjusted *p* value < 0.05 and fold change > 1.5 (Figure 2b, Table S4). The comparisons with most significant differences in expression were between OKO versus YKO, with similar numbers of up‐ and down‐regulated genes (2321 and 2657). In contrast to methylation differences, there was not a significant enrichment of overlap between DEGs from OWT versus YWT and YKO versus YWT. There was an enrichment in age‐associated DEGs between both the GADD45B KO and WT (Figure 2b) and these DEGs overlap with reported aged HSC signature genes (Flohr Svendsen et al. 2021) (Figure S2a). This suggests that though methylation profiles of the YKO were similar to old HSCs, transcriptionally the YKO did not express a more “aged” transcriptome.

IPA upstream pathway analysis of DEGs between comparisons predicts inhibition of RXRA and RXRB in old HSCs compared to young in both WT and KO comparisons, whereas in the YKO versus YWT the reverse is predicted with activation of both RXRA and RXRB in the YKO HSCs (Figure S2b). RXRA and RXRB have been implicated in HSC quiescence (Cabezas‐Wallscheid et al. 2017; Menendez‐Gutierrez et al. 2023; Zeng et al. 2024) with the loss of expression of the retinoic acid receptors leading to decreased quiescence and loss of fitness (Menendez‐Gutierrez et al. 2023). The potential alteration of cell cycle regulation between the YKO and YWT was also predicted in canonical pathway analysis (Figure 2c). These *z*‐scores for the changes in cell cycle in the GADD45B knockouts are largely driven by increased expression of *Cdc25a*, *Cdc25b*, *E2f2*, *E2f4*, and proteasome 20S and 26S subunits in young GADD45B KO HSCs (Table S4). There were no enriched pathways found in the OKO versus OWT DEGs.

To determine if DNAm changes were associated with transcriptional changes, we established the DEGs and overlapping DMR‐NPs from DNAm analysis between comparisons. One hundred and seventy‐six genes were common between DEGs and DMR‐NPs in the OKO versus YKO comparison, 79 in the OWT versus YWT, 153 in the YKO versus YWT, and 5 overlaps were seen in the OKO versus OWT (Figure 2d). We analyzed the correlation when changes were seen in both transcription and DNAm (Figure 2e, Table S5). In general, high levels of DNAm at promoter regions are associated with low expression and conversely, higher levels of gene expression can occur at unmethylated promoters: thus, DNAm changes that regulate gene expression should be inversely correlated (quadrants 2 and 4). However, there was no enrichment for genes in the 2nd and 4th quadrants suggesting most differentially expressed regions were independent of DNAm changes.

### 3.3 Gadd45b‐KO HSCs Do Not Show Any Functional Differences Compared to WT

**FIGURE 3:**
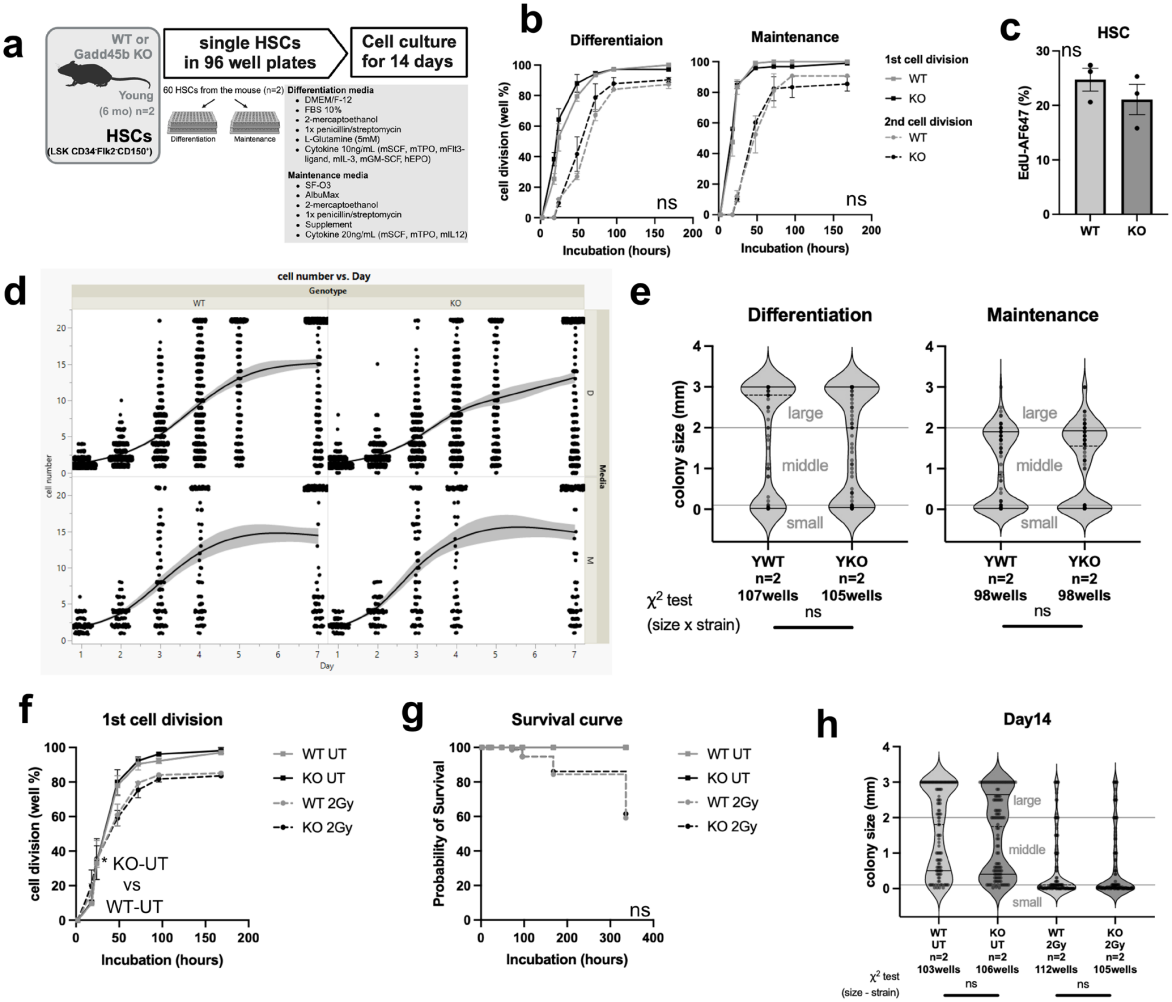
Single cell functional analysis of Gadd45B‐KO and WT HSC (a) Schematic of single cell experiments (b) Initial cell division rate in single HSC cultures (identified as single cells 2 h post plate‐sorting) showing the percentage of wells where cell proliferation was observed for the first (solid line) or second (dotted line) time compared to previously evaluated time point. Two way ANOVA in each time point, in same cell division category. (c) The proliferation frequency of 2000 HSCs from single mice (*n* = 3) cultured in differentiation media with 10 μM EdU for 18 h. Unpaired t test. (d) Trajectories of the cell numbers from single WT or KO HSCs in each medium during the first 7 days of culture (Day 1 is 24 h post sorting). (e) Colony size of cultured young HSCs at Day 14 by culture conditions. Colonies were divided into three groups by size (small (< 0.02 mm), medium (0.02–2 mm), large (> 2 mm)) and tested by chi‐square test in size group and strain. (f) Initial cell division rate with and without DNA damage induction in single young HSC observed as a single HSC 2 h after single‐cell plate sorting. Two way ANOVA in each time point. (g) Survival curve in single HSC culture that were single HSC when observed 2 h after single HSC sorting until 14 days of culture. Log rank test. (h) Colony size of cultured young HSCs at Day 14 with or without DNA damage induction in differentiation media conditions. Colonies were divided into three groups by size (small (< 0.02 mm), medium (0.02–2 mm), large (> 2 mm)) and tested by chi‐square test in size group and strain.

We next examined if methylation or transcriptional alterations in the GADD45B‐KO affected the functional potential of HSCs. As retinoic acid signaling was implicated in both the GADD45B‐KO and aging and GADD45B is an important cell cycle regulator, we initially evaluated the HSCs' cell cycling potential ex vivo. We prepared two types of media for single cell culture: (1) differentiation media, a cytokine rich media with FBS to induce differentiation and proliferation of HSCs and (2) maintenance media, a minimal media without FBS to prolong stem‐cell features ex vivo (Figure 3a). Time course analysis (Figure S3a) of the YKO and YWT HSCs showed no significant differences in the time to first and second cell division in either media condition (Figure 3b). KO HSCs divided slightly faster than WT HSCs, but the difference was not statistically significant. We also examined the proliferation frequency of cells within the first 18 h in culture using EdU incorporation assays on bulk sorted HSCs, but again saw no significant difference between the YKO and YWT HSCs (Figure 3c) or MPPs (Figure S3b). There was also no difference between YWT and YKO when comparing the single cell growth trajectories from Day 1 to Day 7 (Figure 3d) nor were any significant differences seen between the size of the colonies derived from either group of young HSCs at Day 14 (Figure 3e).

Since *Gadd45b* is involved in stress signaling and response to DNA damage, we also induced DNA damage in YKO and YWT HSCs by irradiation to assess the single cell response. Irradiated HSCs had similar division kinetics, regardless of GADD45B expression, and both YKO and YWT HSCs have decreased survival after IR (Figure 3f,g). In irradiated HSCs, there was a decrease in colony size after 7 days of culture that was maintained at 14 days, but with no difference between WT and KO (Figure 3h, Figure S3c). We also did not detect differences in growth kinetics of the old GADD45B‐KO HSCs compared to old WT (Figure S3d). Thus, the loss of GADD45B did not alter the growth kinetics of the stem cells ex vivo.

**FIGURE 4:**
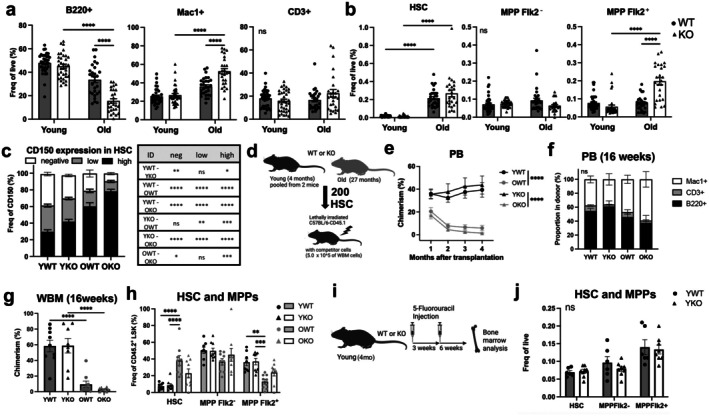
Hematopoietic profiling of young and old Gadd45B‐KO and WT mice. (a) Frequency of B cells (B220^+^), myeloid cells (Mac1^+^) and T cells (CD3^+^) in PB of WT and *Gadd45b* KO mice. (b) Frequency of HSC (LSK[Lin^−^IL‐7Ra^−^Sca‐1^+^c‐kit^+^] CD34^−^Flk2^−^CD150^+^), MPP Flk2− (LSK CD34^+^Flk2^−^) and MPP Flk2+ (LSK CD34^+^Flk2^+^) in BM of WT and *Gadd45b* KO mice. (c) Frequency of lineage biased CD150 subsets of LSKCD34^−^Flk2^−^ cells (d) Experimental design of the competitive transplantation. Two hundred HSCs from the four donor conditions were transplanted into 8 lethally irradiated recipients. (e) Total peripheral blood donor chimerism of donor HSCs. (f) Proportion of B cells, Myeloid cells and T cells in donor derived blood cells. (g) Total bone marrow chimerism 4 months post‐transplant. (h) Frequency of HSC, MPP Flk2^−^ and MPP Flk2^+^ in donor derived LSK BM cells. (i) Experimental design of the 5‐FU injection (j) Frequency of HSC, MPP Flk2^−^ and MPP Flk2^+^ in LSK BM cells at 6 weeks after 2nd 5‐FU injection. Bars indicate mean values for each group and error bars indicate SEM. Two‐way ANOVA was used for statistical analysis. *p < 0.05, **p < 0.005, ***p < 0.0005, ****p < 0.0001, ns, no significant.

To determine if there was an effect of the GADD45B KO on the blood system in vivo, we profiled peripheral blood (PB) and bone marrow (BM) of WT and KO mice both in young and old age by flow cytometry (Figure 4a,b). We demonstrate no significant change in PB in the young KO and WT mice (Figure 4a), but we see more pronounced aging‐associated phenotypes of increased myeloid cells (Mac1^+^) and decreased B‐cells (B220^+^) in the comparisons between OKO versus YKO than in the OWT versus YWT comparison. The frequency of HSC (Lin^−^Sca1^+^cKit^+^ CD34^−^Flk2^−^CD150^+^) increases with age in both WT and KO BM, and there was only a significant change in the frequency in the lymphoid‐biased MPPFlk2^+^ compartment in old KO HSCs (Figure 4b). There is also an exacerbation of the CD150^high^ frequency, the myeloid biased HSCs, increase observed in OKO compared to OWT, with a concomitant decrease in CD150^neg^ (Figure 4c).

To evaluate the functional potential of the HSCs under stress, we performed a competitive transplantation (Figure 4d). All parameters measured for the fitness of the HSCs showed no significant differences between the KO and WT of the same donor age: PB chimerism (Figure 4e), donor lineage output (Figure 4f), BM chimerism (Figure 4g), and donor hematopoietic stem and progenitor composition (Figure 4h). These experiments support that the PB and BM cells of YKO mice are comparable to those of YWT mice, and the functional potential of YKO HSCs is not compromised. In addition, the functional potential of the OKO HSCs did not differ significantly from OWT HSCs (Figure 4f–h), though the composition of the subsets was altered. As an alternative HSC challenge, we injected 5‐Fluorouracil (5‐FU) for two cycles in young KO and WT animals, and after allowing the animals' hematopoietic system to return to homeostasis (6 weeks), we examined the LSK population (Figure 4i). This hematopoietic stress also did not induce differences between the YKO and YWT hematopoietic stem and progenitors (Figure 4j).

### 3.4 DNAm Changes in YKO Do Not Drive Changes in Functional Potential of HSCs

**FIGURE 5:**
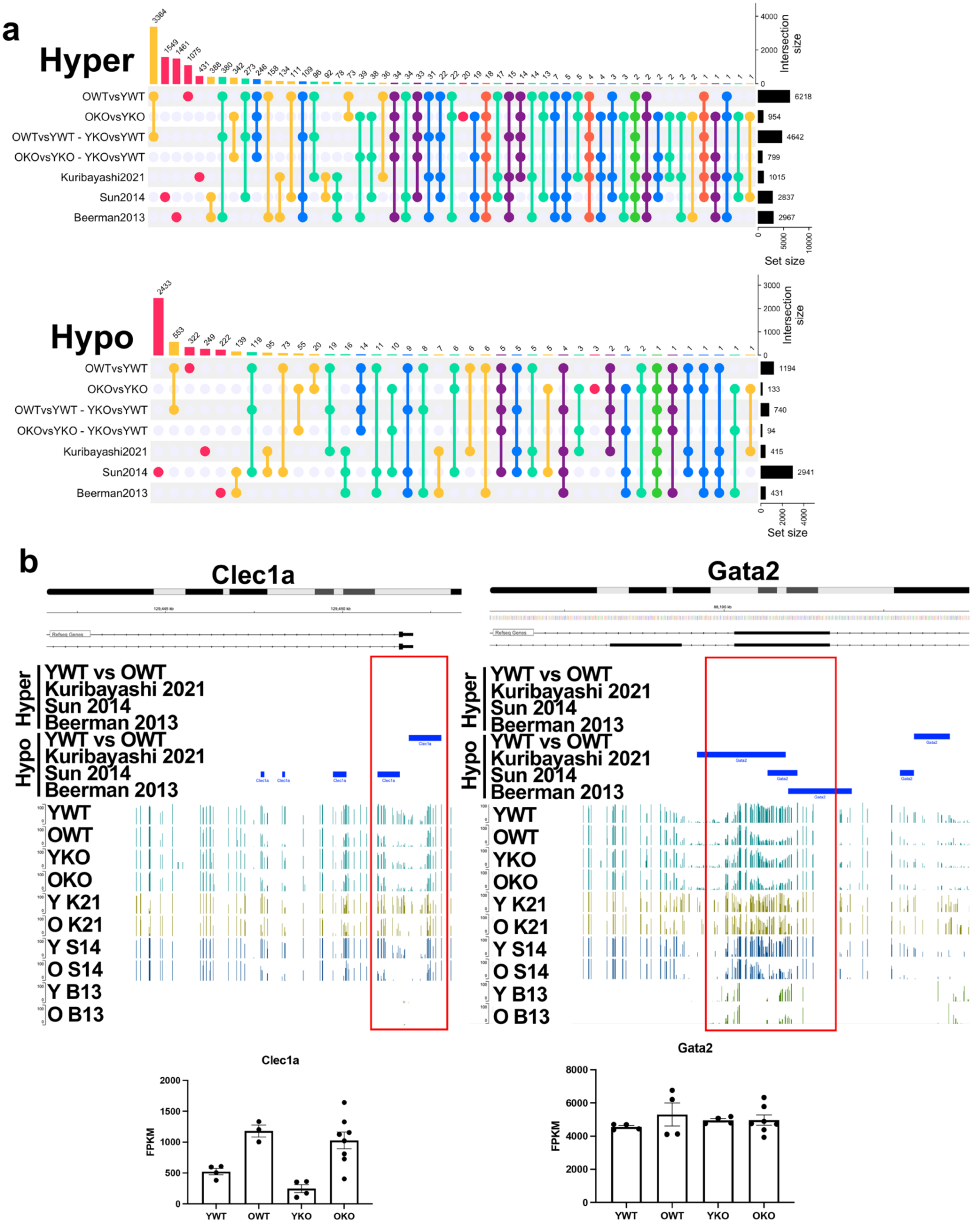
Composite analysis of HSC methylation (a) Upset plot of hyper and hypo DMRs of HSCs from four different studies. The vertical bars indicate the unique number at each combination, and the horizontal bars indicate the total number in each data set. (b, c) Example regions showing DMRs and DNA methylation plotted using Shiny app for (b) *Clec1a* and (c) *Gata2*.

In our analysis, we observed no significant differences in the function of the YKO HSC even though the DNA methylation profiles had significant overlap with aged HSCs. Thus, we wanted to determine if we could narrow the list of DNA methylation changes correlated with functional changes. Towards this, we generated a composite dataset comparing the DNA methylation changes reported in aging HSC studies (Beerman et al. 2013; Kuribayashi et al. 2021; Sun et al. 2014) (Table S6) together with data from the current manuscript. We also included a subset of DNAm changes that we believe to be functionally relevant: age‐associated methylation changes with YKO DMRs removed as they did not lead to functional changes (Figure 5a). Of the DNAm changes associated with WT aging, only 6 DMRs (5 of hyper and 1 of hypo) were common with all three previously reported DMRs. These overlaps were quite low, and since sex, age, coverage differences, and immunophenotype of HSCs between the four data sets could contribute to differences, we generated a searchable method to visualize DNAm reported from each dataset mapped to mm10 (https://niairpnih.shinyapps.io/aging_signature_dna_methylation_analysis/). For instance, *Clec1a*, one of the most reproducible DEG transcripts in aged HSCs, has decreased DNAm at the promoter region of the OHSC in three of the four data sets and has high levels of DNAm in the young Gadd45B‐KO HSCs, but has no coverage of this region in the RRBS dataset (Figure 5b). We find DNAm differences at the start site in the four data sets, though only 2 comparisons define these differences as significant. We also see a decrease in the methylation in young GADD45B‐KO—associated with significant increase in *Clec1a* transcription compared to young WT HSC, but this increase in expression does not contribute to functional differences. We present this tool for searching HSC methylation profiles across multiple experiments.

## 4 Discussion

In this study, we revealed knocking out GADD45B in HSCs induces global increases in DNAm, similar to the increase in DNAm reported in aged HSCs. We found not only did the global increase of DNAm mirror aging phenotypes, but the overall methylation landscapes of the young GADD45B‐KO HSCs were more similar to old HSCs than to young WT HSC. However, these DNAm changes had little effect on functional potential in the assays we performed, which include the gold‐standard competitive transplant assay. One possible reason for this is that GADD45A and GADD45G may have compensatory actions to counteract the loss of GADD45B. Similarly, both GADD45A KO and GADD45G KO mice did not show impaired functional potential of hematopoietic stem and progenitor cells (Chen et al. 2014; Thalheimer et al. 2014). Thus, when one of the *Gadd45* genes is absent, it may be sufficient to induce DNAm changes but not enough to alter the function of the hematopoietic system.

Our integrated analysis of DMR‐NPs and transcriptional changes revealed only limited overlap between methylation and gene expression differences, and no consistent inverse correlation (Figure 2d,e). These findings align with recent studies demonstrating that most age‐associated methylation changes do not directly modulate transcription through promoter methylation (Kuribayashi et al. 2021). Although we focused on DMR‐NPs located near the TSS, these sites did not show robust associations with transcriptional output. Because gene expression is also regulated by enhancer activity, distal regulatory elements, and chromatin architecture, it remains possible that a subset of DNAm changes acts outside promoter regions. These considerations support the interpretation that many of the DNAm alterations shared between GADD45B KO and aged HSC represent non‐functional or distal epigenetic changes that do not drive shifts in HSC functional potential.

DNMT1/3 and TET1/2 are core regulators of DNA methylation maintenance and turnover in HSCs during aging. By comparing our DEG and DMR‐NPs datasets with published DNMT‐ and TET‐deficient hematopoietic stem and progenitor cells datasets, we observed limited overlap at the transcriptional level, whereas a substantially greater degree of convergence was evident at the level of DNA methylation changes (Table S8). This dissociation suggests that Gadd45b primarily influences the epigenetic landscape without directly enforcing the transcriptional programs regulated by DNMT or TET enzymes.

In trying to associate epigenetic and functional changes in whole genome analyses is challenging as there are a vast number of differences defined, and often there is a lack of direct correlation between transcription and changes in DNA methylation. Yu et al. (2016) utilized multicolor lineage tracing to monitor HSC clonal differentiation and found from single cell analysis that epigenetic priming of the stem cells was a better indicator of the functional potential compared to the transcriptome, and that stem cells retained this epigenetic derived potential even when exposed to different environments. We present a model in which HSCs' methylation patterns have been altered (GADD45B‐KO in young HSC), leading to epigenetic changes overlapping with many methylation alterations occurring with aging; however, these overlapping changes do not contribute to altered phenotypes, suggesting these methylation marks are not critical for the changes in functional potential. These data highlight the importance of the methylation sites chosen for epigenetic clocks, as not all age‐associated DNAm changes are direct indicators of functional aging. However, in our analysis using five different epigenetic clocks, all but one clock's predicted age for YKO was similar to YWT, which correlates to the functional potential, suggesting methylation sites for these clocks appear to correlate well with the function of the HSCs, though they may not be directly tied to transcriptional changes. To allow for further studies of the critical alterations in HSCs, we have generated a searchable database for age‐associated methylation changes and defined an abridged library of DNA methylation marks that contribute to the changes in functional potential of HSCs.

## Author Contributions

W.K., M.T.‐Y., H.Y., and F.T.‐T. performed and analyzed the experiments; B.P. performed bioinformatical analyses; I.B., W.K., and M.T.‐Y. designed research and interpreted the results; I.B. and W.K. wrote the manuscript.

## Funding

This work was supported by National Institute on Aging/NIA (AG000992).

## Ethics Statement

All animal care, treatments, and procedures were approved by the NIA Animal Care and Use Committee (ACUC), 469‐TGB‐2025, and were in accordance with the NIH Animal Guidelines.

## Conflicts of Interest

The authors declare no conflicts of interest.

## Supporting information


**Figure S1:** acel70453‐sup‐0001‐FiguresS1‐S4.zip.
**Figure S2:** acel70453‐sup‐0001‐FiguresS1‐S4.zip.
**Figure S3:** acel70453‐sup‐0001‐FiguresS1‐S4.zip.
**Figure S4:** acel70453‐sup‐0001‐FiguresS1‐S4.zip.

**Table S1:** acel70453‐sup‐0002‐TablesS1‐S8.xlsx.
**Table S2:** acel70453‐sup‐0002‐TablesS1‐S8.xlsx.
**Table S3:** acel70453‐sup‐0002‐TablesS1‐S8.xlsx.
**Table S4:** acel70453‐sup‐0002‐TablesS1‐S8.xlsx.
**Table S5:** acel70453‐sup‐0002‐TablesS1‐S8.xlsx.
**Table S6:** acel70453‐sup‐0002‐TablesS1‐S8.xlsx.
**Table S7:** acel70453‐sup‐0002‐TablesS1‐S8.xlsx.
**Table S8:** acel70453‐sup‐0002‐TablesS1‐S8.xlsx.

## Data Availability

At publication, all raw data are available at the NCBI Gene Expression Omnibus database (GEO; http://www.ncbi.nlm.nih.gov/geo/) under the accession number GSE276335. Shiny app is available at https://niairpnih.shinyapps.io/aging_signature_dna_methylation_analysis/.
