# Supplementary material for: Age‐Like Methylation Changes of HSCs in GADD45B Knockout Mice Define Methylation Sites Associated With Loss of Function
Source: Aging Cell. 2026 Mar 20;25(4):e70453. doi: 10.1111/acel.70453 (PMC13140837; doi:10.1111/acel.70453)
Supplement: Supplementary file 1 — Figure S1: acel70453‐sup‐0001‐FiguresS1‐S4.zip. Figure S2: acel70453‐sup‐0001‐FiguresS1‐S4.zip. Figure S3: acel70453‐sup‐0001‐FiguresS1‐S4.zip. Figure S4: acel70453‐sup‐0001‐FiguresS1‐S4.zip. [file ACEL-25-e70453-s002.zip › acel70453-sup-0002-Supinfo2@Supplemental Figure Legends.pdf]

Supplemental Figure 1: Additional DNA methylation analysis of young and old Gadd45B-KO and WT HSC (a) Heatmap of DMCs. Hierarchical clustering was performed using the Ward.D2 method based on Euclidean distance. (b) The definition diagram for DMC, DMR and DMR-NPs. (c) The epigenetic age estimated by 5 independent epigenetic clocks, with actual age shown in samples used for DNAm analysis.

Supplemental Figure 2: Additional Transcriptional analysis of young and old Gadd45B-KO and WT HSC (a) Gene groups whose expression increases or decreases with aging, and overlap with reported aged HSC signature genes (Arthur Flohr Svendsen et al., 2021). (b) IPA analysis of DEGs for the comparisons OWT vs YWT, YKO vs YWT, and OKO vs YKO. No IPA results are shown for OKO vs OWT due to the limited number of DEGs. (c) FPKM value of Gadd45a and Gadd45b. Two-way ANOVA was used for statistical analysis. *:$p<0.05$, **:$p<0.005$, ns: no significant.

Supplemental Figure 3: Single cell culture analysis of Gadd45B-KO and WT HSC (a) Representative images of cells at days 7 and 14 after initiation of single-cell culture. Images with thick borders were taken at 20× magnification; the others were taken at 10× magnification. (b) The proliferation frequency of 2000 MPPs from single mice (n=3) cultured in differentiation media with 10uM EdU for 18 hours. Unpaired - t test. (c) Cell numbers of cultured young HSCs derived from WT and KO mice with or without 2 Gy irradiation, measured on day 7. (d) Trajectories of the cell numbers from single old WT or KO HSCs in each medium during the first 7 days of culture (day 1 is 24 hours post sorting).

Supplemental Figure 4: Gating strategy for flow cytometry analysis of (a) peripheral blood (PB) and **(b)** bone marrow (BM)**.**
