# Supplementary figures and images for: Age‐Like Methylation Changes of HSCs in GADD45B Knockout Mice Define Methylation Sites Associated With Loss of Function
Source: Aging Cell. 2026 Mar 20;25(4):e70453. doi: 10.1111/acel.70453 (PMC13140837; doi:10.1111/acel.70453)

FigS1

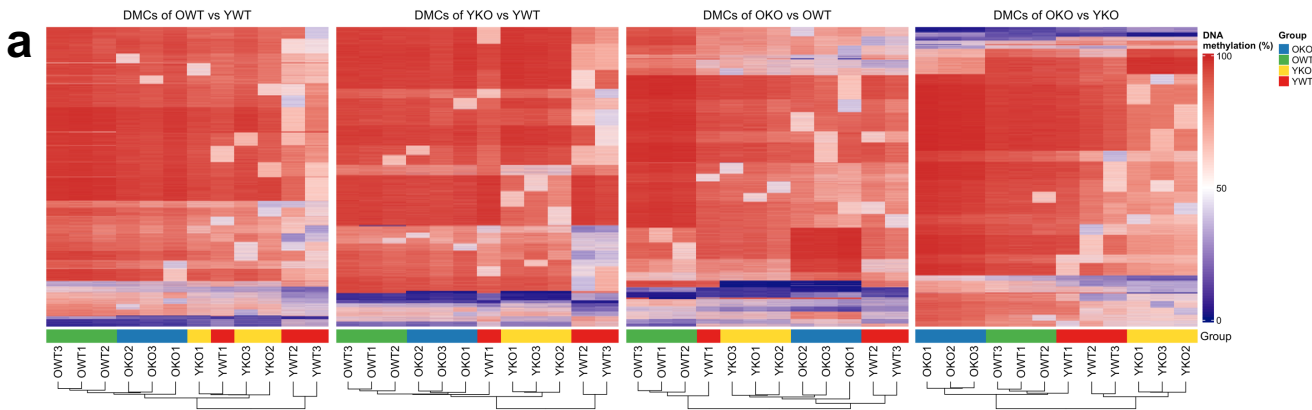

**b**

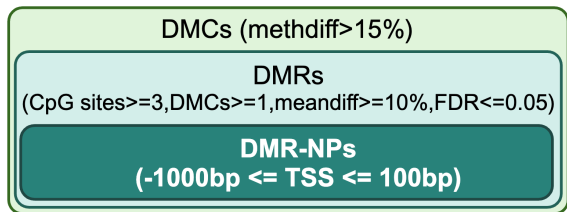

**c**

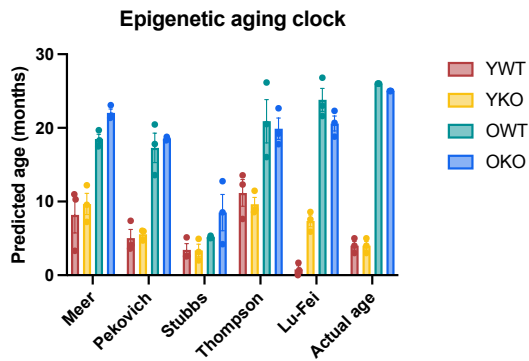

Supplement: Supplementary file 1 — Figure S1: acel70453‐sup‐0001‐FiguresS1‐S4.zip. Figure S2: acel70453‐sup‐0001‐FiguresS1‐S4.zip. Figure S3: acel70453‐sup‐0001‐FiguresS1‐S4.zip. Figure S4: acel70453‐sup‐0001‐FiguresS1‐S4.zip. [file ACEL-25-e70453-s002.zip › acel70453-sup-0003-FigureS1@Figure S1.pdf]

FigS2

a

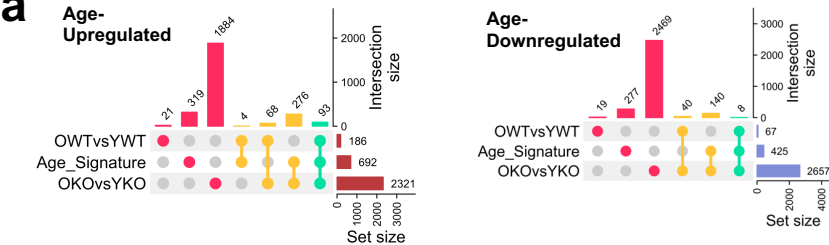

b

OWT vs YWT

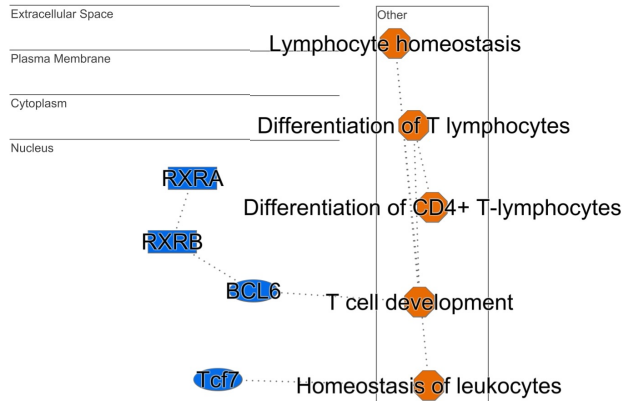

YKO vs YWT

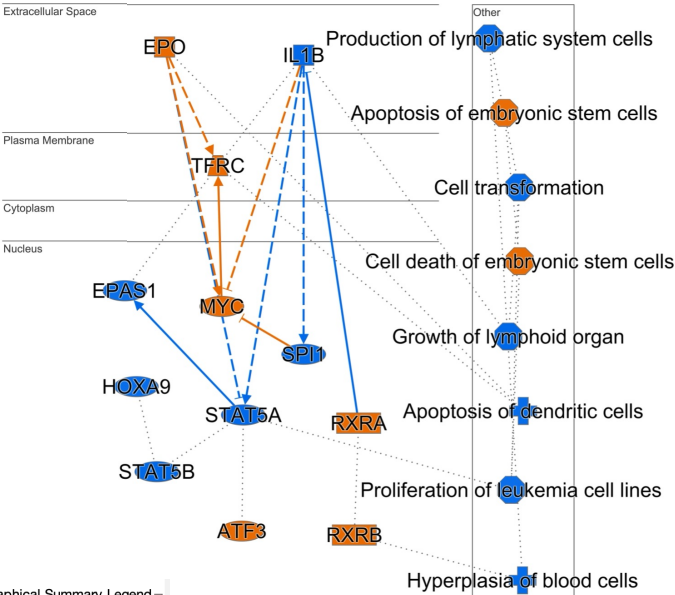

OKO vs YKO

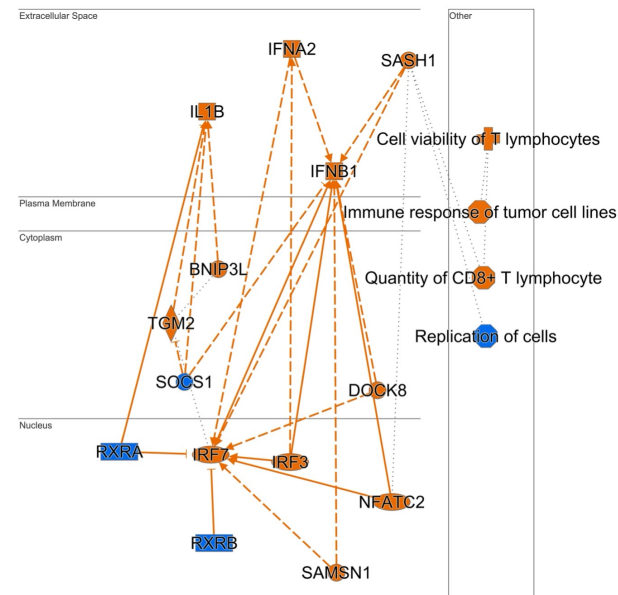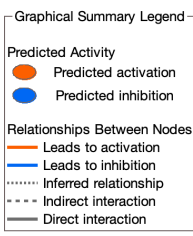

c

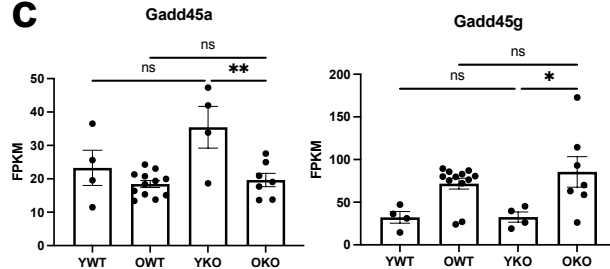

Supplement: Supplementary file 1 — Figure S1: acel70453‐sup‐0001‐FiguresS1‐S4.zip. Figure S2: acel70453‐sup‐0001‐FiguresS1‐S4.zip. Figure S3: acel70453‐sup‐0001‐FiguresS1‐S4.zip. Figure S4: acel70453‐sup‐0001‐FiguresS1‐S4.zip. [file ACEL-25-e70453-s002.zip › acel70453-sup-0004-FigureS2@Figure S2.pdf]

Fig S3

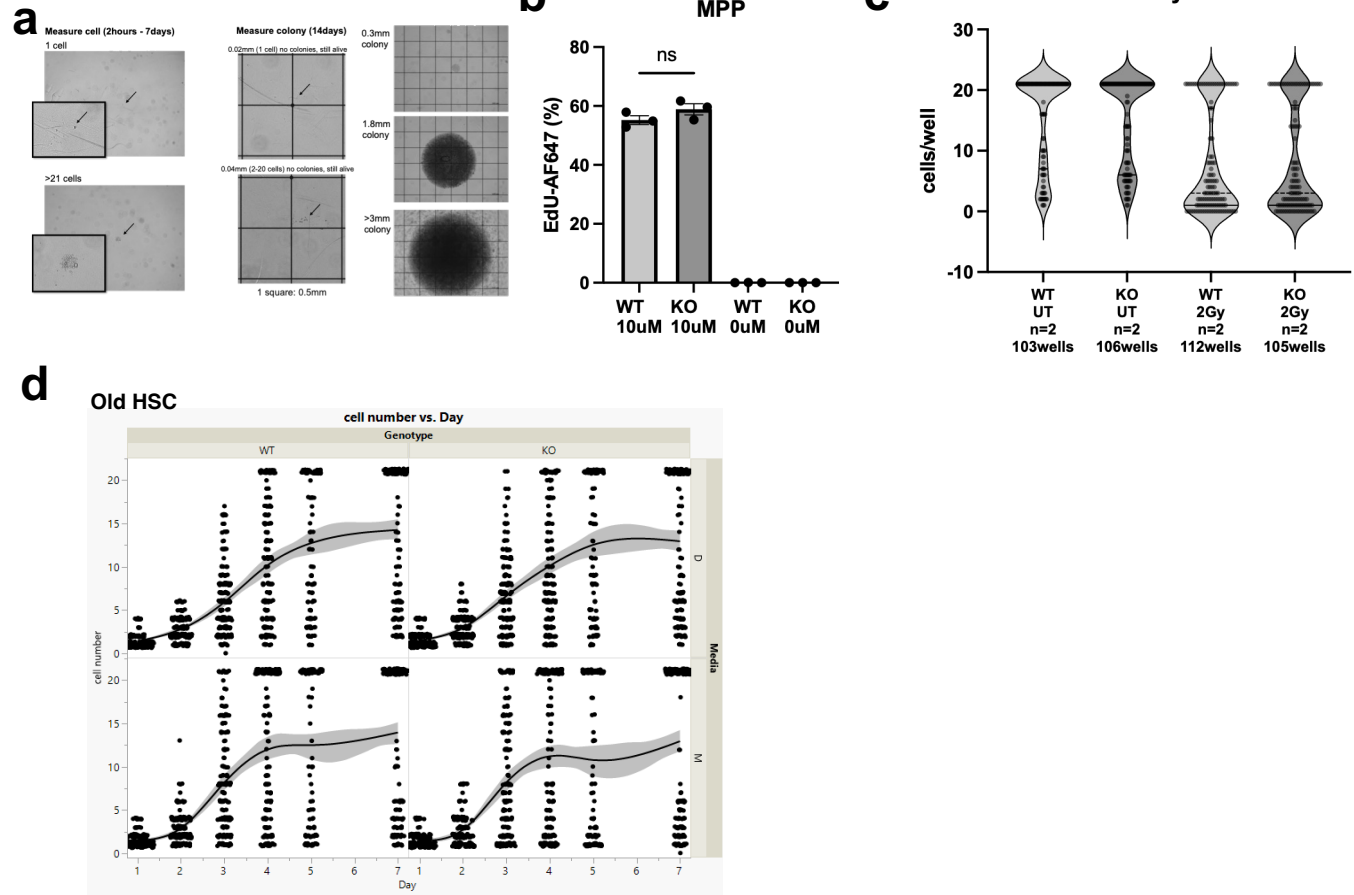

Supplement: Supplementary file 1 — Figure S1: acel70453‐sup‐0001‐FiguresS1‐S4.zip. Figure S2: acel70453‐sup‐0001‐FiguresS1‐S4.zip. Figure S3: acel70453‐sup‐0001‐FiguresS1‐S4.zip. Figure S4: acel70453‐sup‐0001‐FiguresS1‐S4.zip. [file ACEL-25-e70453-s002.zip › acel70453-sup-0005-FigureS3@Figure S3.pdf]

Fig S4

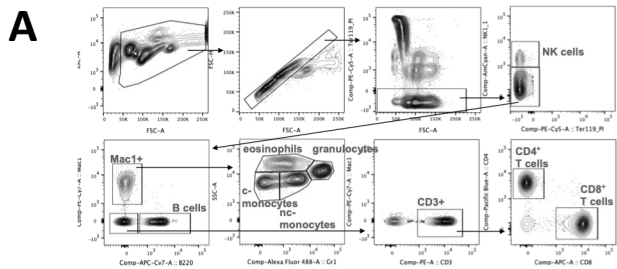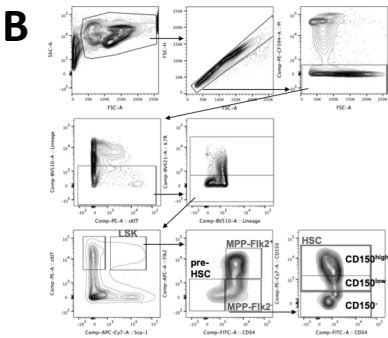

Supplement: Supplementary file 1 — Figure S1: acel70453‐sup‐0001‐FiguresS1‐S4.zip. Figure S2: acel70453‐sup‐0001‐FiguresS1‐S4.zip. Figure S3: acel70453‐sup‐0001‐FiguresS1‐S4.zip. Figure S4: acel70453‐sup‐0001‐FiguresS1‐S4.zip. [file ACEL-25-e70453-s002.zip › acel70453-sup-0006-FigureS4@Figure S4.pdf]
